# Impact of CD68/(CD3+CD20) Ratio at the Invasive Front of Primary Tumors on Distant Metastasis Development in Breast Cancer

**DOI:** 10.1371/journal.pone.0052796

**Published:** 2012-12-26

**Authors:** Noemí Eiró, Iván Pidal, Belen Fernandez-Garcia, Sara Junquera, Maria L. Lamelas, José M. del Casar, Luis O. González, Alfonso López-Muñiz, Francisco J. Vizoso

**Affiliations:** 1 Unidad de Investigación, Fundación Hospital de Jove, Gijón, Asturias, Spain; 2 Servicio de Ginecología, Fundación Hospital de Jove, Gijón, Asturias, Spain; 3 Servicio de Cirugía General, Fundación Hospital de Jove, Gijón, Asturias, Spain; 4 Servicio de Anatomía Patológica, Fundación Hospital de Jove, Gijón, Asturias, Spain; 5 Departamento de Morfología y Biología Celular, Facultad de Medicina, Universidad de Oviedo, Oviedo, Asturias, Spain; Health Canada, Canada

## Abstract

Tumors are infiltrated by macrophages, T and B-lymphocytes, which may favor tumor development by promoting angiogenesis, growth and invasion. The aim of this study was to investigate the clinical relevance of the relative amount of macrophages (CD68^+^), T-cells (CD3^+^) and B-cells (CD20^+^) at the invasive front of breast carcinomas, and the expression of matrix metalloproteases (MMPs) and their inhibitors (TIMPs) either at the invasive front or at the tumor center. We performed an immunohistochemical study counting CD3, CD20 and CD68 positive cells at the invasive front, in 102 breast carcinomas. Also, tissue sections were stained with MMP-2, -9, -11, -14 and TIMP-2 antibodies, and immunoreactivity location, percentage of reactive area and intensity were determined at the invasive front and at the tumor center. The results showed that an increased CD68 count and CD68/(CD3+CD20) ratio were directly associated with both MMP-11 and TIMP-2 expression by mononuclear inflammatory cells at the tumor center (p = 0.041 and p = 0.025 for CD68 count and p = 0.001 and p = 0.045 for ratio, respectively for MMP-11 and TIMP-2). In addition, a high CD68/(CD3+CD20) ratio (>0.05) was directly associated with a higher probability of shortened relapse-free survival. Multivariate analysis revealed that CD68/(CD3+CD20) ratio was an independent factor associated with distant relapse-free survival (RR: 2.54, CI: (1.23–5.24), p<0.01). Therefore, CD68/(CD3+CD20) ratio at the invasive front could be used as an important prognostic marker.

## Introduction

Development of an invasive cancer is not only the result of genetic changes in tumor cells but also the result of the interplay between tumor and stromal cells [1]. Tumors are infiltrated by a large number of immune cells that constitute the main cell population of tumor microenvironment, where they can account for up to 50% of the total tumor mass in invasive breast carcinomas. Historically, tumor-infiltrating leukocytes have been considered as an intrinsic defensive mechanism against developing tumors [2]–[3]. However, increasing evidence indicates that leukocyte infiltration may favor tumor development by promoting angiogenesis, growth, and invasion [4]–[5]. This may be due to inflammatory cells that probably influence cancer promotion by secreting cytokines, growth factors, chemokines and proteases, which stimulate proliferation and invasiveness of cancer cells [6]–[8].

Inflammatory cells have gained a renewed interest in breast cancer research due to our increased understanding of their role in tumor development, and also due to our increased ability to identify each cell type. Leukocyte infiltrate includes a variable representation of leukocytes, including macrophages, neutrophils, mast cells, and T and B-lymphocytes [4], [9]. There are evidences indicating that different types of breast carcinomas may have different types of leukocyte infiltrate with distinct abilities to control tumor growth according to their tumor dissemination. Thus, whereas macrophages are known to have several pro-tumor functions and macrophage infiltration has also been associated with worse prognosis [4], [10]–[11], it has been reported that both T- and B-lymphocytes perform an important immunological response by inhibiting cancer development and progression [12]–[20].

Metastasis development is regulated not only by intrinsic genetic changes in malignant cells, but also by the tumor microenvironment. Matrix metalloproteases (MMPs) play an essential role in the degradation of the stromal connective tissue and basement membrane components, which are key elements in tumor invasion and metastasis. In fact, in the metastatic process across the axillary lymph node chain in breast cancer, MMP-1 expression by mononuclear inflammatory cells (MICs) from the sentinel lymph node (SLN) was significantly associated with metastatic spread to non-SLNs [21]. MMPs cleave proapoptotic factors and induce a more aggressive phenotype generating apoptotic resistant cells [22], and also regulate cancer-related angiogenesis, both positively through their ability to mobilize or activate proangiogenic factors [23], or negatively through the generation of angiogenesis inhibitors, such as angiostatin and endostatin [24]. The activity of MMPs is specifically inhibited by the so-called tissue inhibitors of metalloproteases (TIMPs). In previous reports we analyzed the expression of several MMPs and TIMPs (MMP-1, 2, 7, 9, 11, 13 and 14, and TIMP-1, 2 and 3), either at the invasive front or at the tumor center of breast carcinomas, in many of the women included in the present study [25]–[28]. Thus, we identified a phenotype of MICs characterized by the expression of specific MMPs and TIMPs (MMP-2, 9 11 and 14, and with TIMP-2) in the tumor center, associated with distant metastasis development [25]–[26], suggesting that inflammatory cells at the invasive front can polarize their phenotype impacting on tumor progression [27]. These tumors also showed an up-regulation of inflammatory-related genes (IL-1, -5, -6 and -17, IFNβ and NFkB), which emphasize their importance in promoting disease metastasis and recurrence [29].

Considering that the invasive front is the area where some of the most important interactions between cancer cells and tumor supporting stroma take place [30], we investigate the relevance of the relative amount of macrophages (CD68), T-cells (CD3) and B-cells (CD20) in this tumor location from breast carcinomas. Also, we study their relationship with MMPs and TIMPs expression, either at the invasive front or at the tumor center. Thus, we found that a high CD68/(CD3+CD20) ratio (>0.5) at the invasive front is associated with tumor aggressiveness and poor prognosis in patients.

## Materials and Methods

### Ethics Statement

Women were treated according to the guidelines used in our Institution (Hospital de Jove). Written informed consent, approved by “Hospital de Jove Ethics and Investigation Committee”, was obtained from all patients before the evaluation of tumor samples. The study adhered to National regulations and was approved by our Institution's Ethics and Investigation Committee.

### Patient selection, characteristics and tissue specimen handling

This study comprises 102 women with a histological confirmed diagnosis of early invasive breast cancer and treated between 1990 and 2003. Many of these women have been included in previous studies of our group [25]–[28]. We selected women with the following inclusion criteria: invasive ductal carcinoma and a minimum of 5 years of follow-up for those women without tumor recurrence. The exclusion criteria were the following: metastatic disease at diagnosis, prior history of any kind of malignant tumor, bilateral breast cancer at diagnosis, have been treated with any type of neoadjuvant therapy, development of loco-regional recurrence during the follow-up period or development of a second primary cancer. From patients fulfilling these criteria, we randomly selected a sample size of 102 patients in accordance to 4 different groups stratified with regard to nodal status and to the development of metastatic disease, which were the key measure variables of the study. Thus, we included an important number of cases in both node-positive and node-negative patient subgroups in order to guarantee the statistical power of the survival analysis. Patient characteristics included in the two main groups, with or without distant metastases, are listed in Table 1. Menopausal status was defined as “postmenopausal” if 1 year was elapsed since the last menstrual period. For reporting the Histological Grade we used the Nottingham combined histologic grade (Elston-Ellis modification of Scarff-Bloom-Richardson grading system) [31].

**Table 1:** Basal characteristics of 102 patients with invasive ductal carcinoma of the breast.

CHARACTERISTICS	Without recurrence No. (%)	With recurrence No. (%)
**Total cases**	59 (100)	43 (100)
**Menopausal status**		
Premenopausal	18 (30.5)	12 (27.9)
Postmenopausal	41 (69.5)	31 (72.1)
**Tumoral size**		
T1	31 (52.5)	19 (44.2)
T2	28 (47.5)	24 (55.8)
**Nodal status**		
N (−)	28 (47.5)	12 (27.9)
N (+)	31 (52.5)	31 (72.1)
**Histological grade**		
Well Dif. (I)	20 (33.9)	7 (16.3)
Mod. Dif. (II)	31 (52.5)	16 (37.2)
Poorly Dif. (III)	8 (13.6)	20 (46.5)
**Nottingham prognostic index**		
<3.4	25 (42.4)	8 (18.6)
3.4–5.4	25 (42.4)	22 (51.2)
>5.4	9 (15.3)	13 (30.2)
**Estrogen Receptor**		
Negative	16 (27.1)	23 (53.5)
Positive	31 (52.5)	18 (41.9)
**Progesterone Receptor**		
Negative	20 (33.9)	27 (62.8)
Positive	27 (45.8)	14 (32.6)
**Adjuvant radiotherapy**		
No	44 (74.6)	21 (48.8)
Yes	15 (25.4)	22 (51.2)
**Adjuvant systemic therapy**		
Chemotherapy	18 (30.5)	18 (41.9)
Tamoxifen	24 (40.7)	9 (20.9)
Chemotherapy plus sequential Tamoxifen	10 (16.9)	7 (16.3)
No treatment	7 (11.9)	9 (20.9)
**HER2 Status**		
Negative	49 (83.1)	36 (83.7)
Positive	8 (13.6)	7 (16.3)
**Basal like phenotype**		
Non basal like	30 (50.8)	23 (53.5)
Basal like	15 (25.4)	18 (41.9)

The end-point of our study was distant metastatic relapse. The median follow-up period in patients without metastases was 85 months, and 52 months in patients with metastases.

### Tissue arrays and immunohistochemistry

Breast carcinoma tissue samples were obtained at the time of surgery. Samples were removed from the tumors, avoiding grossly necrotic tissues, routinely fixed, paraffin-embedded and stored. Histopathological representative tumor areas of invasive front and tumor center were defined in hematoxylin and eosin-stained sections and marked on the slide. The invasive front was defined as the tumor advancing edge, which corresponds to a 2 mm margin surrounding the tumor and containing cancerous cells, and the tumor center was defined as the tumor area inside the invasive front. Tumor tissue microarray (TMA) blocks containing primary tumor samples were performed as described previously [25]. We analyzed 2 cores of the invasive front and 2 cores of the tumor center in each case (double redundancy) as it has been demonstrated to correlate properly with conventional immunohistochemical staining methods [25], [27].

Four composite high-density TMA blocks were performed, consecutively cut in 5 µm sections with a microtome (Leica Microsystems GmbH, Wetzlar, Germany) and transferred to adhesive-coated slides. One section from each TMA block was stained with hematoxylin and eosin, and these slides were then reviewed to confirm that the sample was representative of the invasive front and tumor center of the original tumor. Immunohistochemistry was performed using a TechMate TM50 autostainer (Dako, Glostrup, Denmark), where sections were incubated with the following antibodies (ready to use): CD3 (T-lymphocytes), CD20 (B-lymphocytes) and CD68 (macrophages) all purchased from Dako (Glostrup, Denmark).

In previous reports from our group, we found a specific MICs phenotype characterized by high MMP-2, 9, 11, 14, and TIMP-2 expression, which correlated significantly with distant metastasis development [25]–[28]. Consequently, in the present study we performed a new staining set using antibodies against these specific proteins, in the tissue arrays from the invasive front and those from the tumor center. Antibodies for MMPs and TIMPs were purchased from Neomarker (Lab Vision Corporation, Fremont, CA, USA), and the dilution used was: 1/50 for MMP-2, -14 and TIMP-2; 1/100 for MMP-9; and 1/200 for MMP-11. To enhance antigen retrieval, tissue sections were treated in a PT-Link® (Dako) at 97°C for 20 min, in citrate buffer of pH 6.1 for MMP-14, in EDTA buffer of pH 9 for TIMP-2. Antibodies for MMP-2, -9 and -11 do not require antigen retrieval. The negative control was DakoCytomation mouse or rabbit serum diluted at the same concentration as the primary antibody. All the dilutions were made in Antibody Diluent, (Dako, Glostrup, Denmark) and incubated 30 min at room temperature.

Endogenous peroxidase activity was blocked by incubating the slides in peroxidase-blocking solution (Dako) for 5 min. The EnVision Detection Kit (Dako) was used as the staining detection system. Sections were counterstained with hematoxylin, dehydrated with ethanol, and permanently coverslipped.

### Immunohistochemistry analysis

Five fields per core, corresponding to areas of higher immunostaining and without necrosis, were evaluated with a 400× power objective, counting CD3, CD20 and CD68 positive cells, in 1 mm^2^ final area, at the invasive front. If there was no tumor sample in a particular core, 10 fields were then evaluated in another one in order to obtain the same final area. We obtain a total score and this is the value of CD3, CD20 or CD68 for each tumor.

For each MMP or TIMP antibody studied, we determined the immunoreactivity location, percentage of reactive area and intensity, at the invasive front and at the tumor center. An image analysis system composed of the Olympus BX51 microscope, digital camera system DP12 and soft analysis (analySIS®, Soft Imaging System, Münster, Germany) was used in the tumor sections (stained with antibodies and counterstained with hematoxylin), as described before [32]. To evaluate immunostaining intensity we used a numeric score ranging from 0 to 3, reflecting the intensity as follows: 0, no reactivity; 1, weak reactivity; 2, moderate reactivity; and 3, intense reactivity. Using an Excel spreadsheet, the mean score was obtained by multiplying the intensity score (I) by the percentage of reactivity area (PA) and the results were added together (total score: I×PA). This overall score was then averaged with the number of cores performed for each patient. If there was no tumor in a particular core, then no score was given. In addition, the mean score of two core biopsy samples was calculated for each tumor. This scoring evaluation was based on a global evaluation of staining areas corresponding to tumor cells as well as to stromal cells. Nevertheless, in the present work we also evaluated the immunohistochemical staining exclusively for mononuclear inflammatory cells (MICs).

### Statistical analysis

Differences in percentages were calculated with the chi-square test. Immunostaining score values for each protein were expressed as a median (range). Correlation between score values was calculated by using the Spearman correlation test. Comparison of immunostaining values between groups was made with the Mann-Whitney or Kruskall-Wallis tests. Statistical results were corrected applying Bonferroni's correction. For relapse-free survival analysis we used the Cox's univariate method. Cox's regression model was used to examine interactions between different prognostic factors in multivariate analysis. Only parameters that achieve statistical significance for distant relapse-free survival in the univariate analysis were included in the multivariate analysis. The PASW Statistics 18.0 software (SPSS Inc, Chicago, IL, USA) was used for all calculations. *p*<0.05 was considered as significant.

## Results

**Figure 1:**
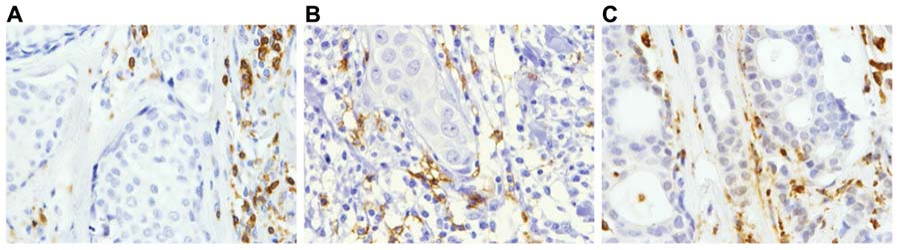
Representative examples of immunohistochemical stainings at the invasive front from breast carcinomas (×200 magnification). (A) Membranous staining of CD3 indicating T-lymphocytes. (B) Membranous staining of CD20 indicating B-lymphocytes. (C) Cytoplasmic staining of CD68 indicating macrophages.

**Figure 2:**
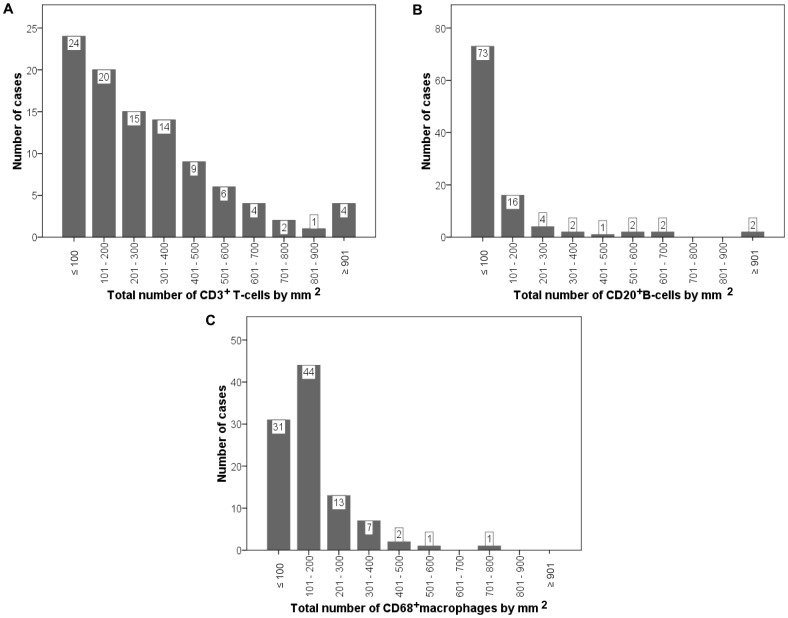
Distribution of the total number of CD markers by mm^2^ at the invasive front, in 102 breast carcinomas. CD3 (A), CD20 (B) and CD68 (C).

Immunostainings for CD3, CD20 and CD68 were performed in TMA blocks from invasive ductal carcinoma of the breast (Figure 1), showing a membranous staining for CD3 and CD20, whereas CD68 staining is found in the cytoplasm. Our results demonstrate a wide variability among tumors in the number of CD3^+^ T-cells (median: 214.00 (0–999), CD20^+^ B-cells (29.50 (0–1152) or CD68^+^ macrophages (141.00 (14–727), by 1 mm^2^ at the invasive front (Figure 2). We found direct correlations between the number of CD68^+^ macrophages and the number of CD3^+^ T-cells (r sub S = 0.57; p = 0.0001) or the number of CD20^+^ B-cells (r sub S = 0.51; p = 0.0001), and specially between the number of CD3^+^ T-cells and the number of CD20^+^ B-cells (r sub S = 0.71; p = 0.0001).

We examined the possible relationship between the overall number of intratumoral MICs at the invasive front, or the relative ratio of these cells [number of CD68^+^ macrophages/number of lymphocytes (number of CD3^+^ T-cells+number of CD20^+^ B-cells), further named as CD68/(CD3+CD20) ratio], and the clinico-pathological characteristics of patients and tumors (Table 2). Our results demonstrated a direct relationship between the number of CD3^+^ T-cells and premenopausal status (p = 0.009); whereas this same cell count was inversely associated with both ER^+^ and PgR^+^ status (p = 0.04 and p = 0.003, respectively). The number of CD20^+^ B-cells was directly associated with HER2^+^ status (p = 0.009). The number of CD68^+^ macrophages was inversely associated with PgR^+^ status and directly associated with HER2^+^ status (p = 0.027). However, our results showed no significant association between the CD68/(CD3+CD20) ratio and any clinico-pathological characteristics (Table 2).

**Table 2:** Relationship between inflammatory cells count or ratio and clinico- pathological characteristics in 102 patients with invasive ductal carcinoma of the breast.

CHARACTERISTICS	No.	CD3	CD20	CD68	CD68/(CD3+CD20)
		median (range)	median (range)	median (range)	median (range)
**Total cases**	102	214 (0–999)	29 (0–1152)	141 (14–727)	0.5 (0–6.6)
**Menopausal status**		***p*** ** = 0.009**			
Premenopausal	30	322 (9–999)	50 (0–1121)	158 (31–404)	0.3 (0.1–5.4)
Postmenopausal	72	167 (0–987)	18 (0–1152)	128 (14–727)	0.5 (0–6.6)
**Tumoral size**					
T1	50	207 (0–987)	22 (0–1152)	128 (15–727)	0.5 (0–5.4)
T2	52	242 (12–999)	34 (0–1121)	154 (14–577)	0.6 (0.1–6.6)
**Nodal status**					
N (−)	40	201 (9–987)	27 (0–1152)	136 (15–727)	0.5 (0.1–6.3)
N (+)	62	250 (0–999)	32 (0–1121)	142 (14–577)	0.6 (0–6.6)
**Histological grade**					
Well Dif. (I)	27	197 (9–987)	25 (0–1152)	140 (15–727)	0.5 (0.1–5.4)
Mod. Dif. (II)	47	228 (12–999)	30 (0–1121)	142 (49–577)	0.6 (0.1–6.6)
Poorly Dif. (III)	28	252 (0–542)	35 (0–156)	139 (14–416)	0.6 (0–5.4)
**Nottingham prognostic index**					
<3.4	33	172 (9–954)	7 (0–655)	122 (15–727)	0.5 (0.1–5.4)
3.4–5.4	47	267 (0–999)	41 (0–1152)	143 (21–577)	0.5 (0–6.6)
>5.4	22	250 (14–756)	40 (0–252)	170 (14–416)	0.5 (0.1–4.0)
**Estrogen Receptor**		***p*** ** = 0.040**			
Negative	39	298 (0–987)	41 (0–1152)	181 (14–727)	0.6 (0–4)
Positive	49	151 (9–895)	10 (0–1121)	122 (34–362)	0.6 (0.1–6.3)
**Progesterone Receptor**		***p*** ** = 0.003**		***p*** ** = 0.002**	
Negative	47	267 (27–987)	40 (0–1152)	182 (14–727)	0.6 (0.1–6.1)
Positive	41	144 (0–895)	10 (0–1121)	105 (35–314)	0.6 (0–6.3)
**HER2 Status**			***p*** ** = 0.009**	***p*** ** = 0.027**	
Negative	85	209 (0–999)	16 (0–1152)	137 (14–727)	0.5 (0–6.6)
Positive	15	359 (36–917)	101 (0–576)	186 (54–577)	0.6 (0.1–1.1)
**Basal like phenotype**					
Non basal like	53	197 (9–895)	14 (0–1121)	137 (34–577)	0.7 (0.1–6.3)
Basal like	33	251 (0–987)	40 (0–1152)	142 (14–727)	0.4 (0–4.0)

Mann-Whithney or Kruskall-Wallis tests.

We had previously identified a significant percentage of tumors with a MICs phenotype characterized by a molecular profile with specific MMPs and TIMPs increased expression, and associated with a high metastatic rate [25]–[28]. Thus, in the present work we determined the expression of these significant proteins (MMP-2, 9, 11, 14, and TIMP-2) in the tumor samples, and analyzed the possible relationship between the presence of different MICs phenotypes at the invasive front, and MMPs and TIMPs expressions by tumors both in the invasive front and in the tumor center.

With regard to global expression (score values) of MMPs and TIMPs, our result showed a direct correlation between MMP-2 score values and CD3 (r = 0.21, p = 0.038), CD20 (r = 0.25, p = 0.011) or CD68 (r = 0.32, p = 0.001) counts at the invasive front; whereas MMP-9 score values correlated with CD68 count (r = 0.21, p = 0.041) in this same tumor location. On the other hand, TIMP-2 score values at the tumor center correlated inversely with CD3 (r = −0.23, p = 0.021) or with CD20 (r = −0.21, p = 0.036) count in the invasive front, but correlated directly with CD68/(CD3+CD20) ratio in this same tumor location (r = 0.24, p = 0.014).

**Figure 3:**
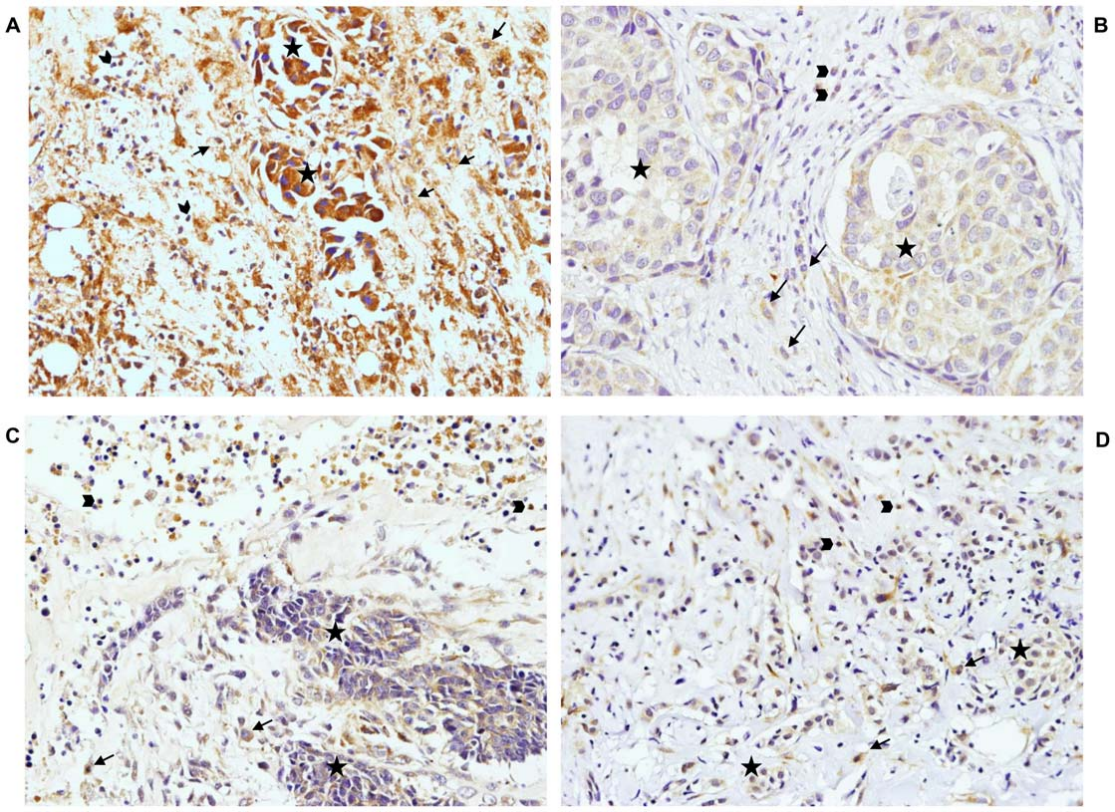
Representative example of immunostaining. MMP11 (A) and TIMP2 (B) immunostaining at the tumor center and MMP9 (C) and MMP14 (D) at the invasive front (×200 magnification), indicating the different cell types. Tumor cells (★), lymphocytes (<$>\raster(70%)="rg2"<$>) and macrophages (<$>\raster(70%)="rg1"<$>).

Figure 3 shows examples of immunostaining for different MMPs and TIMPs, at tumor center and at the invasive front. We found several significant associations between the different MICs counts at the invasive front and the expression of MMPs and TIMPs by MICs from the invasive front or from the tumor center (Table 3). Thus, high CD3, CD20 or CD68 counts were significantly associated with MMP-9 expression, at the invasive front; whereas high CD68 count was significantly associated with MMP-14 and TIMP2 in this same tumor location. Also, we found that high CD68 count and CD68/(CD3+CD20) ratio were associated with both MMP-11 and TIMP-2 expressions by MICs at the tumor center. In addition, it is interesting our finding indicating that if there is a high CD68/(CD3+CD20) ratio at the invasive front, most of MICs with a positive MMP-11 or TIMP-2 phenotype at the tumor center are macrophages (Figure 3A and B, respectively). In this figure, MMP-11 staining demonstrates that apart from tumor cells with large nucleus and an intense cytoplasmic staining, there are a small number of lymphocytes with rounded nucleus surrounded by a small positive cytoplasm, but the most abundant cells type in the tumor center are macrophages, which are the large, round cells that contain a central round nucleus and an abundant clear positive cytoplasm.

**Table 3:** Relationship between inflammatory cells count or ratio at invasive front and MMPs/TIMPs expression by mononuclear inflammatory cells at invasive front or tumor center.

	MICs at invasive front											
	MMP-9			MMP-11			MMP-14			TIMP-2		
	−	+	*p*	−	+	*p*	−	+	*p*	−	+	*p*
**CD3**	185	327	**0.006**	209	266.5	N.S	210	261	N.S	154	317.5	**0.001**
	(0–987)	(76–999)		(9–954)	(0–999)		(0–999)	(12–542)		(0–895)	(27–999)	
**CD20**	15.5	85	**0.029**	25	38.5	N.S	23	55.5	N.S	8	74.5	**0.002**
	(0–1152)	(0–576)		(0–655)	(0–1152)		(0–1152)	(0–302)		(0–1121)	(0–1152)	
**CD68**	130.5	184	**0.036**	128	166	N.S	132.5	186.5	**0.015**	118	178.5	**0.002**
	(14–727)	(15–416)		(21–727)	(14–577)		(15–727)	(14–577)		(21–416)	(14–727)	
**CD68/(CD3+CD20)**	0.6	0.43	N.S	0.5	0.48	N.S	0.45	0.7	N.S	0.6	0.43	N.S
	(0–6.6)	(0.1–0.9)		(0.1–5.4)	(0–6.6)		(0–6.1)	(0.2–6.6)		(0–6.6)	(0.1–6.1)	

	MICs at tumor center											
	MMP-9			MMP-11			MMP-14			TIMP-2		
	−	+	*p*	−	+	*p*	−	+	*p*	−	+	*p*
**CD3**	209	256	N.S	222.5	199	N.S	209	234	N.S	210	252.5	N.S.
	(0–999)	(36–451)		(0–999)	(9–542)		(12–999)	(0–917)		(0–999)	(9–954)	
**CD20**	25	75	N.S	29.5	29	N.S	30	21	N.S	22.5	35.5	N.S.
	(0–1152)	(0–211)		(0–1152)	(0–211)		(0–1152)	(0–576)		(0–1152)	(0–664)	
**CD68**	140	167	N.S	130.5	166.5	**0.041**	143	137	N.S	128	184.5	**0.025**
	(14–727)	(62–577)		(14–727)	(40–577)		(15.727)	(14–577)		(14–577)	(40–727)	
**CD68/(CD3+CD20)**	0.5	0.6	N.S	0.44	0.9	**0.001**	0.43	0.6	N.S	0.4	0.7	**0.045**
	(0–6.6)	(0.2–1.1)		(0–6.61)	(0.2–6.3)		(0.1–6.6)	(0–6.3)		(0–5.37)	(0.1–6.6)	

Mann-Whithney test. MICs: mononuclear inflammatory cells. Data are expressed as median (range). N.S: not significant.

**Figure 4:**
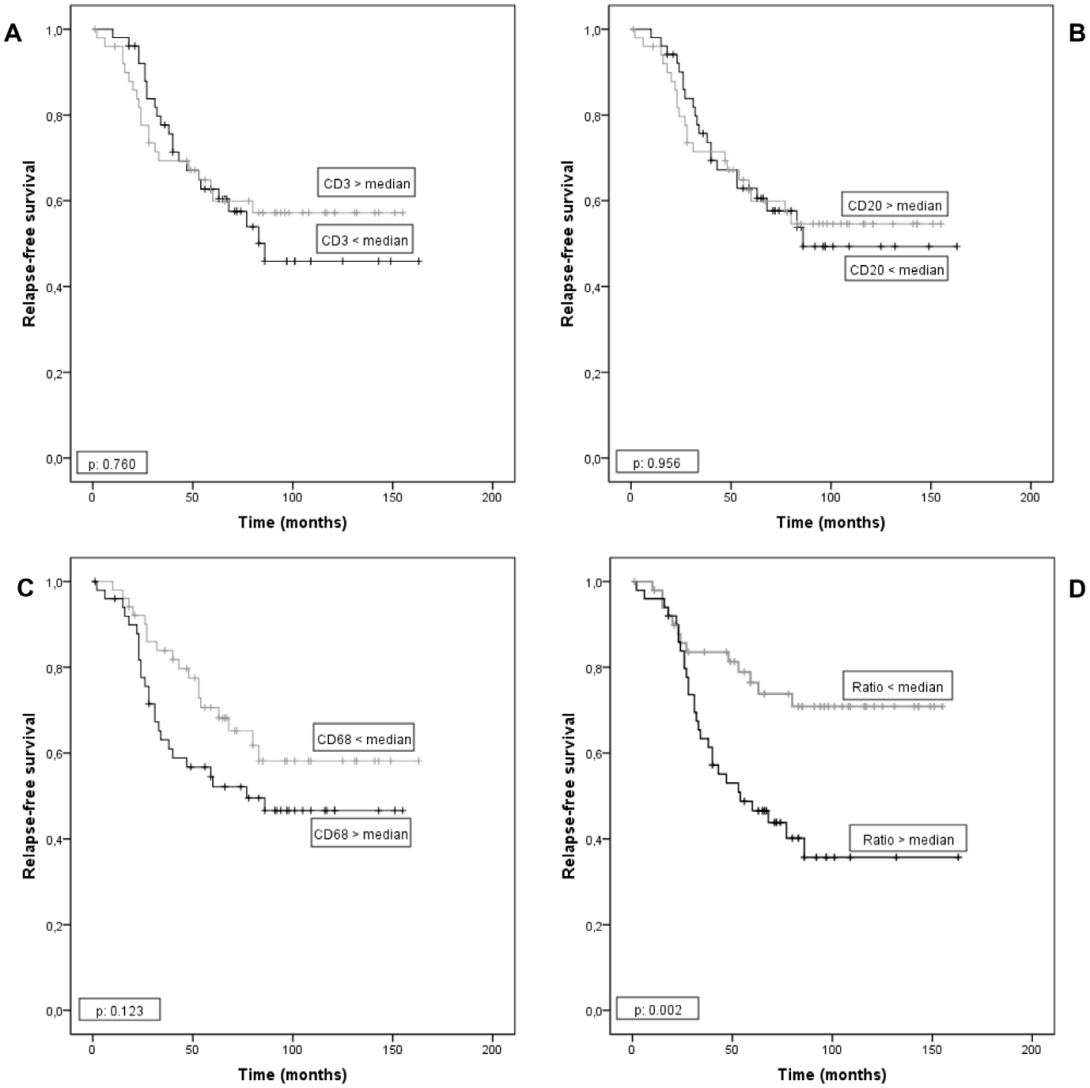
Probability of relapse-free survival as a function of CD markers count for 102 patients with invasive ductal carcinoma. CD3 count (A), CD20 count (B), CD68 count (C) and CD68/(CD3+CD20) ratio (D).

The possible influence of the number of the different inflammatory cell types on relapse-free survival was evaluated in all patients included in the present study. For this purpose, we took the corresponding median value of the total number of each cell type by 1 mm^2^ at the invasive front as cut-off point. Univariate analysis indicates that CD3, CD20, or CD68 count showed no significant associations with relapse-free survival (Figure 4). Nevertheless, our results showed that a high CD68/(CD3+CD20) ratio was significantly associated with a higher probability of shortened relapse-free survival (p = 0.002) (Table 4 and Figure 4D). Multivariate analysis according to Cox's model demonstrated that tumor stage (II: (relative risk (RR) (confidence interval (CI) = 1.8(0.7–4.5); III: 4.6(1.8–12.0); *p* = 0.003) and PgR status (positive: 0.4(0.2–0.8), *p* = 0.011) were significant and independently associated with distant relapse-free survival. Nevertheless, this same analysis also demonstrated that CD68/(CD3+CD20) ratio was significant and independently associated with distant relapse-free survival (Table 4).

**Table 4:** Cox's univariate (HR) and multivariate (RR) analysis of the significant relationships between MMPs, TIMPs expression or CD68/(CD3+CD20) ratio at the tumor center or at the invasive front, and relapse-free survival.

Tumor location	Factor	No. of patients	Event frequency	HR (95% CI)	RR (95% CI)
**TUMOR CENTER**	**TIMP2**				
	Score < median vs. >median	51/51	9/34	4.62 (2.21–9.65)****	3.23 (1.51–6.92)***
	MIC (−) vs. (+)	72/30	20/23	3.77 (2.06–6.89)****	4.37 (2.31–8.25)****
	**MMP11**				
	MIC (−) vs. (+)	76/26	18/25	9.19 (4.73–17.85)****	8.80 (4.40–17.61)****
**INVASIVE FRONT**	**MMP9**				
	Score < median vs. >median	50/49	16/25	2.03 (1.08–3.80)*	2.22 (1.15–4.29)*
	MMP14				
	MIC (−) vs. (+)	74/24	24/17	3.38 (1.81–6.31)****	3.41 (1.75–6.63)****
	**TIMP2**				
	MIC (−) vs. (+)	49/50	15/26	1.89 (1.01–3.58)*	2.51 (1.28–4.92)**
	**CD68/(CD3+CD20) Ratio**	51/50	13/29	2.68 (1.39–5.17)***	2.54 (1.23–5.24)**

Abbreviations: MIC: mononuclear inflammatory cells; HR: hazard ratio; RR: relative risk; CI: confidence interval.

* p<0.05;

** p<0.01;

*** p<0.005;

**** p<0.001.

## Discussion

Inflammation is now considered a hallmark of cancer and can play a role in all aspects of tumor biology, including initiation, promotion, angiogenesis, and metastasis [4], [27], [33]–[34]. It is known that the activation of oncogenes can trigger the production of inflammatory molecules and the recruitment of inflammatory cells. But the potential effects of the inflammatory cell infiltrate in breast cancer seem to be diverse and complex. Therefore, in this study we investigate the impact of different inflammatory cell types at the invasive front from breast carcinomas on distant metastasis development. We consider that this is of special interest because the invasive front is the area where some of the most important interactions between cancer cells and the tumor supporting stroma take place [30]. Our results showed a biological heterogeneity among breast tumors with regard to these cellular infiltrates at the invasive front. In addition, we found that a high CD68/(CD3+CD20) ratio at the invasive front is significant and independently associated with the occurrence of distant metastasis.

There are data indicating that, depending on the cell type present and their functional profile, inflammatory cells can either suppress or promote tumor growth. We analyzed the expression profile of the individual inflammatory cell types, and our results are in accordance with other studies indicating that tumor-infiltrating lymphocytes correlate with hormone receptor-negative or HER2^+^ status, or with high grade/highly proliferative tumors, although we did not find correlation with favorable long-term prognosis [12]–[19]. In addition, it has been reported that activated B cells can mediate tumor regression by itself and confers host T-cell antitumor immunity. Likewise, it was suggested that effector B cells can serve as a useful adjunct in adoptive T-cell therapy [35].

Tumor-associated macrophages arise from circulating monocytes that migrate into tissues in response to chemical signals and differentiate into macrophages. In breast cancer, macrophages have been found to comprise up to 50% of the breast tumor mass [36]. Tumor-associated macrophages produce a variety of cytokines and chemokines, as well as growth factors for both epithelial and endothelial cells, which play a key role in tumor growth and metastasis [4], [10]–[11]. Our results are in accordance with previous studies reporting an association between macrophages density and PgR^−^ or HER-2^+^ status [37]. However, also in accordance with Mahmoud *et al.*, we found that overall macrophage numbers are not related to prognosis in breast cancer in a multivariate analysis [37]. This may be due the density of macrophages was correlated with higher tumor grade in the present study as well as in previous studies [37]–[39]. Hence, multivariate analysis is thus essential when examining the relation between macrophage infiltration and survival. Nevertheless, this latter analysis led us to identify a high CD68/(CD3+CD20) ratio was a potent independent factor for predicting distant metastasis relapse-free survival in our patient population. Therefore, we describe here, for the first time, a study evaluating the relative amount of different MICs at the invasive front in breast carcinomas, using a new ratio that correlates with patient survival and could be useful in predicting patient outcome. We consider this is a relevant finding since the role of inflammatory cells in cancer seems to be complex, and this ratio can reflect a more objective result of the interactions between both anti-tumor and pro-tumor effects of the different inflammatory cells.

The end point of the present study was the occurrence of distant metastasis, which is regulated not only by intrinsic genetic changes in malignant cells, but also by the microenvironment. MMPs play an essential role in tumor invasion and metastasis via degradation of the stromal connective tissue and basement membrane components, and are inhibited by TIMPs. In previous reports we identify a phenotype of MICs characterized by the expression of specific MMPs and TIMPs at the tumor center, and associated with distant metastasis development [25]–[28], which also showed an up-regulation of inflammatory-related genes [29]. According to this, in the present study we determined the expression of these significant proteins (MMP-2, 9, 11, 14, and TIMP-2) in those breast cancer samples and analyzed the possible relationship between the different inflammatory cells counts at the invasive front and the expression of MMPs and TIMPs, either at the invasive front or at the tumor center. Then, we found several associations between the inflammatory cell types and some of these factors. Nevertheless, the most relevant finding was the association between high CD68/(CD3+CD20) ratio and the expression of MMP-11 (stromalysin-3) or TIMP-2 by the MICs at the tumor center. This is a relevant finding considering that both MMP-11 and TIMP-2 are the two principal factors defining the pro-metastatic phenotype of MICs in our previous studies [25]–[28]. Therefore, these results may indicate that a high CD68/(CD3+CD20) ratio at the invasive front contributes to polarize macrophages to achieve a high metastatic phenotype at the tumor center. In addition, it is remarkable our finding indicating that if there is a high CD68/(CD3+CD20) ratio at the invasive front, most of MICs with a positive MMP-11 or TIMP-2 phenotype at the tumor center are macrophages.

A limitation of the present study was the lack of a complete study of the count for the different MICs at the tumor center. It was due to the absence of enough tissue sample in many cases, because of their utilization in our previous expression studies on MMPs, TIMPs and other factors in breast carcinomas. Nevertheless, we observed that most of MICs in tumor center have macrophage-like morphology, indicating an important contribution of these stromal cells to tumor biology in this tumor location.

In summary, our results contribute to characterize the inflammatory cell infiltrate in breast cancer, and their relationship with prognostic evaluation and MMPs/TIMPs expression. Further studies will be necessary to assess if this CD68/(CD3+CD20) ratio at the invasive front can contribute to identify patients with breast cancer candidates to different therapeutic strategies based on immuno-modulation. In fact, several strategies against tumor-associated macrophages have already been published [40]–[42], and several reports indicate the effectiveness of activated B-cells in cellular immunotherapy of malignancies [43]–[46]. Hence, to design breast tumor immunotherapy and vaccine strategies hereafter, it will be necessary to consider humoral immunity in addition to the cell mediated immunity, as a potential therapeutic tool.
